# Sisal-Fiber-Reinforced Polypropylene Flame-Retardant Composites: Preparation and Properties

**DOI:** 10.3390/polym15040893

**Published:** 2023-02-10

**Authors:** Zhenhua Wang, Weili Feng, Jiachen Ban, Zheng Yang, Xiaomin Fang, Tao Ding, Baoying Liu, Junwei Zhao

**Affiliations:** 1 College of Chemistry and Chemical Engineering, Henan University, Kaifeng 475004, China; 2 Institute of Science and Technology, Minsheng College, Henan University, Kaifeng 475004, China

**Keywords:** polypropylene, sisal fiber, flame retardant, preparation, mechanical properties

## Abstract

Natural-fiber-reinforced polypropylene (PP) composites with a series of advantages including light weight, chemical durability, renewable resources, low in cost, etc., are being widely used in many fields such as the automotive industry, packaging, and construction. However, the flammability of plant fiber and the PP matrix restricts the application range, security, and use of these composites. Therefore, it is of great significance to study the flame retardants of such composites. In this paper, sisal-fiber-reinforced polypropylene (PP/SF) flame-retardant composites were prepared using the two-step melt blending method. The flame retardant used was an intumescent flame retardant (IFR) composed of silane-coated ammonium polyphosphate (Si-APP) and pentaerythritol (PER). The influence of different blending processes on the flammability and mechanical properties of the composites was analyzed. The findings suggested that PP/SF flame-retardant composites prepared via different blending processes showed different flame-retardant properties. The (PP/SF)/IFR composite prepared by PP/SF secondary blending with IFR showed excellent flame-retardant performance, with a limited oxygen index of about 28.3% and passing the UL-94 V-0 rating (3.2 mm) in the vertical combustion test. Compared with the (PP/IFR) /SF composite prepared by a matrix primarily blended with IFR and then secondly blended with SF, the peak heat release rate (pk HRR) and total heat release (THR) of the (PP/SF)/IFR composite decreased by 11.3% and 13.7%, respectively. In contrast, the tensile strength of the (PP/SF)/IFR system was 5.3% lower than that of the (PP/IFR)/SF system; however, the overall mechanical (tensile, flexural, and notched impact) properties of the composites prepared using three different mixing processes were similar.

## 1. Introduction

In order to target the global warming threat, the world is committed to controlling peak carbon emissions in the hope of becoming carbon neutral in the near future. Manufacturers need greater awareness of their production chains and need to develop new materials with reusable or renewable natural resources. Regarding this, natural-plant-fiber-reinforced thermosetting and thermoplastic composite materials are rapid developments in basic research and industrial applications as they are created to be light, renewable, chemically resistant, low-cost, easy to manufacture, completely or partially recyclable, and biodegradable [1,2,3]. Sisal fiber (SF)-reinforced polypropylene (PP) composite (PP/SF) materials have been widely used in automotive applications because of the advantages they impart in low cost and density, as well as satisfactory mechanical properties coupled with processing [4,5,6]. However, the inherent flammability of the PP matrix and SF has limited the applications of their composites for many semi-structural components that require strict fire safety [5,6,7]. In this regard, it is of great importance to study the fire performance of natural-fiber-reinforced polymeric composites.

In recent years, flame retardant research on PP has mainly been based on the addition of the intumescent flame retardant (IFR) system composed of ammonium polyphosphate (APP) and pentaerythritol (PER), so as to further optimize the flame-retardant effect. The main research has focused on the following: (1) The microencapsulation of APP to improve the poor compatibility between APP and substrate, low flame retardant efficiency, and portability of APP [8,9]. (2) The search for an efficient carbon source to improve the carbonization [10,11]. (3) The adoption of a new acid source to replace APP [12,13,14]. (4) The compounds of different flame retardants [15,16,17,18,19]. (5) The development of one-component flame retardants [20,21,22]. The flame-retardant modification of natural fibers is mainly achieved by the flame retardant post-treatment of fibers or fabrics [23,24,25,26,27]. To address the flammability of the PP/SF composite, flame retardant is recommended for the composite to constrain and delay the spread of fire after ignition. Among the kinds of flame retardants, the current popular flame retardant system applied to PP/SF is the compound system composed of phosphorous flame retardants, among which the more classic combustible formula is APP as the main flame retardant and PER or metal hydroxide as the synergist [5,6,7,28,29].

Processing technology has a great influence on the physical and mechanical properties of SF-reinforced polymeric composites due to the fiber impregnation and mixing, final length and diameter, and interface adhesion being closely related to the processing variables [30,31,32]. The existing research has mainly focused on how to control the production quality of these kinds of composite materials in different manufacturing technologies, such as extrusion, compression molding, resin transfer molding, etc. [33,34]. However, studies on the effect of processing technology on the flame retardancy and mechanical properties of the PP/SF composite is still scarce, and there is still a gap in further research on the effect of processing technology on the flame-retardant mechanism of PP/SF composite during combustion. In light of this, flame retardant PP/SF composites were prepared with an intumescent flame retardant system consisting of silane-coated ammonium polyphosphate (Si-APP) and PER in this work, and the influence of the blending process on the flame retardancy and mechanical properties of PP/SF composites was studied; the corresponding influencing mechanism was also analyzed.

## 2. Materials and Methods

### 2.1. Materials

Homopolymerized polypropylene pellets (PP, T03, density 0.9 g/cm^3^, melt flow index 3.0 g/10 min, melting point temperature 168.5 °C) were purchased from Dongguan Juzhengyuan Technology Co., Ltd., Dongguan, China. Sisal fiber (SF) roving yarn, comprising a bundle of elementary fibers without surface chemical treatment, was provided by Guangxi Longzhou Strong Hemp Industry Co., Ltd., Chongzuo, China. Ammonium polyphosphate with silane structure (Si-APP, TF-201W, phosphorus content ≥31%, nitrogen content ≥14, average degree of polymerization ≥1000) was purchased from the company of Shifang Taifeng new flame retardant, Shifang, China [35]. Pentaerythritol (PER) was supplied by Tianjin Kemiou, Chemical Reagent Co., Ltd., Tianjin, China.

### 2.2. Sample Preparation 

#### 2.2.1. Experimental Technological Process

**Figure 1:**
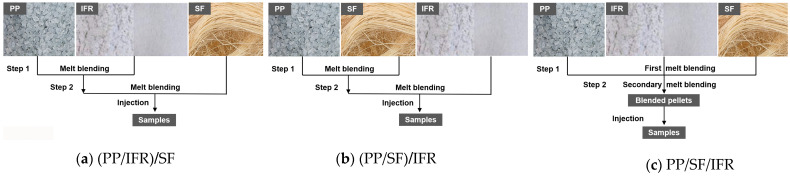
Flow chart of sample preparation of PP/SF flame-retardant composites.

The two-step melt blending method was used for the preparation of PP/SF flame-retardant composites. SF and IFR were mixed with a PP matrix in a different order, as shown in Figure 1. After each melt blending step, the extruded strips were cut into pellets and dried for subsequent processing. In order to better distinguish each sample, the abbreviations of the composite materials prepared under different mixing processes were simply named as (PP/IFR)/SF, (PP/SF)/IFR, and PP/IFR/SF, where (PP/IFR)/SF indicates that the matrix and flame retardant were firstly melt blended, and then remixed with SF (as shown in Figure 1a). (PP/SF)/IFR indicates that the matrix and SF were firstly melt blended, and then remixed with IFR (as shown in Figure 1b). PP/IFR/SF indicates that PP, IFR, and SF were firstly melt blended, followed by a second blend (as shown in Figure 1c).

#### 2.2.2. Preparation of PP/SF Flame-Retardant Composites

Sisal fibers were cleaned with distilled water to ensure the removal of any surface impurities, and then dried at 80 °C in an oven for 12 h. The dried sisal fibers were soaked in sodium hydroxide solution with a weight percentage of 10 wt% for 4 h and washed with distilled water until neutrality; we then put them in the oven at 80 °C for 12 h to dry.

The PP pellets, Si-APP, and PER were dried at 80 °C in an oven for 8 h to remove any hygroscopic moisture. According to the previous research experience of our research group [36], all of the raw materials were configured according to the formula in Table 1 for melt blending. The auxiliaries here refer to the processing auxiliaries including compatibilizers and antioxidants. The equipment used for blending was a corotating twin screw extruder (AK22, Nanjing Keya Chemical Complete Equipment Company, Jiangsu, China), the extrusion temperature was from 178 °C to 180 °C, and the screw speed was 80 rpm. The standard samples for flammability and mechanical tests were injection molded from 178 °C to 180 °C using a UN90A2 injection machine (Guangdong Yizumi Precision Machinery Co., Ltd., Guangdong, China). The injection pressure and pressure holding time were 55 bar and 10 s, respectively. The prepared samples were annealed in a drying oven at 80 °C for 4 h to be tested.

### 2.3. Characterization

The combustion behaviors of samples were conducted using a cone calorimeter (Fire Testing Technology Ltd., East Grinstead, UK) under an external heat flux of 50 kW/m^2^ in accordance with ISO 5660-1. The specimen size used was 100 × 100 × 6 mm^3^.

A fire oxygen index apparatus (TTech-GBT2406-1, Testech Testing Instrument Technology Co., Ltd., Suzhou, China) was used for the limiting oxygen index (LOI) tests, according to the GB/T 2406.2-2009 standard [35]. The tests were conducted using rectangular samples (80 mm × 10 mm × 4 mm). The LOI value of the composite was the average of the minimum oxygen concentration, which supports ten replicate samples which retained combustion.

The Underwriters Laboratories-94 (UL-94) vertical burning tests were conducted using a vertical burning tester (TTech-GBT2408 [35], Testech Testing Instrument Technology Co., Ltd., Suzhou, China). The tests were performed according to the GB/T 2408-2008 standard with samples of size 125 mm × 12.5 mm × 3.2 mm. The final UL-94 rating of the composite was judged by the total flame burning time of five samples.

Tensile and flexural tests were performed on a universal testing machine (TCS-2000, GOTECH Testing Machines Inc., Qingdao, China), along with a ZBC-8400 impact testing machine (MTS Industrial System Co., Ltd., Hong Kong, China) for the notched Izod impact. All of the tests were performed at room temperature. The tensile test was conducted and evaluated according to the GB/T 1040-2006 standard [35], with measurements conducted with a crosshead speed of 50 mm/min. Flexural tests (according to the GB/T9341-2008 standard [35]) were performed at the rate of 2 mm/min with a span distance of 64 mm. The notched Izod impact behaviors of the composites were measured according to the national GB/T 1043-2008 standard [35]. All of the mechanical test results given represented an average value of at least five tests.

The thermal stability of the composites was analyzed via thermogravimetric tests (TGA) conducted using a TGA/SDTA851e Thermogravimetric Analyzer (Mettler-Toledo International Co., Ltd., Zurich, Switzerland). The measurements were conducted from room temperature to 800 °C with a heating rate of 10 °C/min under a protective atmosphere of nitrogen.

The fracture surface after the notched impact test and charred residue obtained from the cone test of the PP/SF composites were observed using a field emission scanning electron microscope (JSM-7610F, Electronics Corporation, Tokyo, Japan). Furthermore, a laser Raman spectrometer (Renishaw inVia, Gloucestershire, UK) with a laser excitation source of 780 nm was used for the detection of the internal structure of residual carbon after vertical burning tests. 

## 3. Results and Discussion

### 3.1. Flammability of PP/SF Flame-Retardant Composites

The properties of polymer composites are closely related to their morphology and structure. Different processing technology will affect the internal structure of polymer products, and then affect the performance of products. Therefore, the effects of different feeding processes on the mechanical properties and flame retardancy of PP/SF flame-retardant composites were investigated. The results of the LOI test and vertical burning test of pure PP and PP/SF composites are presented in Table 1. The (PP/IFR)/SF and PP/IFR/SF samples had a high burning rate and could not reach any UL-94 rating. Pure PP is very flammable and its limiting oxygen index value is only 18.4%. The addition of sisal fiber and flame retardant can effectively improve the flammability of PP, which showed that the LOI value increased by at least 37.2%. Compared with (PP/IFR)/SF, the LOI value of PP/IFR/SF composites increased to 25.7%. The difference in LOI values may be caused by the different dispersion of flame retardants at the matrix and fiber interface. The (PP/SF)/IFR composite reached a UL-94 V-0 rating, showing excellent fire resistance, and a remarkably high LOI value of 28.3% was obtained, which increased by 14.6% compared with the (PP/IFR)/SF system.

**Figure 2:**
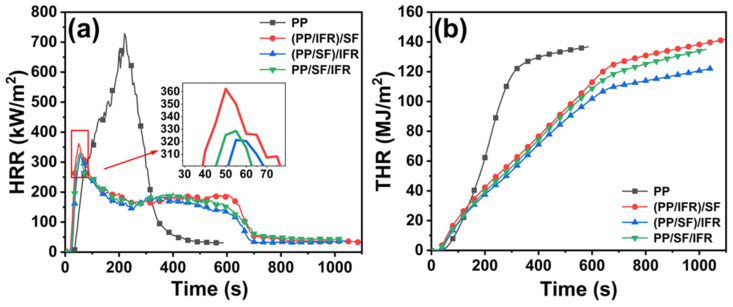
HRR (**a**) and THR (**b**) curves of pure PP and PP/SF flame-retardant composites.

When the sample is burned under the thermal radiation of the conical electric heater, the flame will consume a certain concentration of oxygen in the air and release a certain calorific value of combustion. The combustion behavior of PP and PP/SF composites was evaluated using a conical calorimeter. The experimental principle was to calculate and measure the heat release rate, mass loss rate, and other parameters in the combustion process according to the amount of oxygen consumed by materials during combustion, and then analyze and judge the combustion performance of the material. The results of the cone calorimeter experiment are listed in Table 2. Figure 2 shows the curves of the heat release rate (HRR) and the total heat release (THR) versus time. In general, compared with pure PP, the addition of flame retardants shortened the time to ignition (TTI) of the PP/SF composites, with the peak heat release rate (pk HRR) decreasing and the total smoke production (TSP) increasing, which indicated that the combustion process of the PP/SF composites was suppressed and incomplete. The pk HRR and THR of the PP/SF flame-retardant composites obtained in different blending sequences were different. The pk HRR of (PP/SF)/IFR and PP/IFR/SF were 11.3% and 9.3%, lower than that of (PP/IFR)/SF. Compared with (PP/IFR)/SF, the THR value of (PP/SF)/IFR decreased to 122.2 MJ/m^2^ and reduced by 13.7%. These results showed that the (PP/SF)/IFR composite exhibited better fire resistance, which was consistent with the LOI and vertical combustion test results.

Multiple heat release peaks appeared in the release rate curves of composites under different processes. According to the analysis of the mechanism of flame retardants during combustion [35], Si-APP can play its flame retardant role via both the condense phase and the gas phase. Si-APP was decomposed by heat during combustion and produced active free radicals such as PO· and PO_2_· into the gas phase, which can trap active radicals such as H·, O· and HO· in the flame reaction, so as to inhibit the combustion reaction. In the condensed phase, Si-APP was dehydrated and decomposed by heating, and the first exothermic peak was produced (as shown in Figure 2a). As the combustion process went on, the phosphoric acid substances generated via Si-APP thermal decomposition promoted the dehydration of the matrix and SF into carbon residues; in this way, a dense charred layer was formed onto the underlying substrate surface to isolate the internal structure from air and heat, further inhibiting the combustion process. The residual charred layer and the protected matrix further decomposed to form a more stable charred layer structure, resulting in the emergence of a second exothermic peak, as seen in Figure 2a.

According to the above research results, although the total amount of flame retardant added to the matrix was the same, different processing sequences lead to different flame-retardant effects. The composite, with PP and SF blended first, and then mixed with flame retardants, shows better flame retardancy.

### 3.2. Morphology of Char Residues

**Figure 3:**
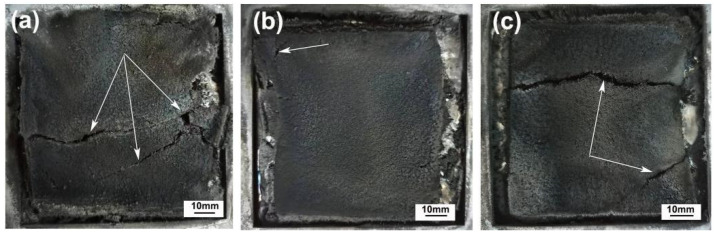
Digital photographs of charred residue of PP/SF composites after cone calorimeter test: (**a**) (PP/IFR)/SF; (**b**) (PP/SF)/IFR; (**c**) PP/IFR/SF.

**Figure 4:**
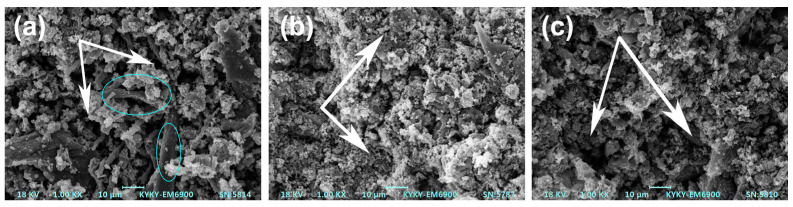
SEM images of charred residue of PP/SF composites after cone calorimeter test. (**a**) (PP/IFR)/SF; (**b**) (PP/SF)/IFR; (**c**) PP/IFR/SF.

The quality of residual carbon is closely related to the flame retardant efficiency. Figure 3 and Figure 4 show the digital photos of the residual carbon layer of composite material after the cone calorimeter test and SEM photos, respectively, from which the carbon formation of the flame retardant system after combustion can be directly reflected. As can be seen from Figure 3, although the carbon layer of the composites obtained by different blending processes was relatively complete, cracks were not absent (as indicated by the arrow). There were many cracks on the charred layer of the (PP/IFR)/SF composite. The incomplete and fragmented charred layer provides channels for combustible gas, heat, and oxygen to enter the underlying matrix, resulting in flame retardant failure.

In contrast, the charred layer of PP/IFR/SF and (PP/SF)/IFR composites was relatively thick and dense. In particular, the charred layer formed by (PP/SF) /IFR was thick and continuous, which could effectively play the role of heat and oxygen isolation, so as to endow the composite with excellent flame-retardant performance.

The internal structure of the carbon layer was further analyzed via SEM (as shown in Figure 4). The (PP/IFR)/SF composite formed a fluffy and porous charred layer after combustion, and only part of the surface of sisal fibers was covered by the charred residue (as shown in Figure 4a). There were numerous holes and cracks along the interface between fibers and the matrix (as indicated by the ellipse), which provided channels for heat and the gases that support combustion. The charred layer produced by the PP/IFR/SF composite was relatively dense, and the sisal fibers were mostly coated by the charred layer (as shown in Figure 4c), which endowed the PP/IFR/SF composite with a better flame-retardant effect. The charred layer of the (PP/SF)/IFR composites was relatively compact, with fewer cavities and good quality (as indicated by the arrow in Figure 4b). The dense charred layer can prevent the contact between volatile combustible gas and external oxygen.

**Figure 5:**
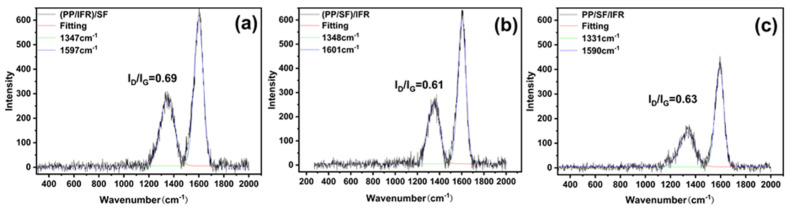
Raman spectra of the char residue of PP/SF flame−retardant composites. (**a**) (PP/IFR)/SF; (**b**) (PP/SF)/IFR; (**c**) PP/IFR/SF.

The quality of the carbon layer is not only related to its external morphology, but also to its internal graphitization degree. Raman spectroscopy was used to evaluate the graphitization degree of charred residue formed during combustion after the cone calorimeter test; the corresponding results are shown in Figure 5. The D-peak and G-peak are two characteristic peaks of the carbon atomic crystal near 1300 cm^−1^ and 1580 cm^−1^, representing a lattice defect of the carbon atom and the in-plane stretching vibration of the sp^2^ hybridization of the C atom, respectively [37,38]. The ratio of the integrated intensities of D and G bands (I_D_/I_G_) is used to estimate the graphitization degree of the charred residue [39]. In general, the smaller the I_D_/I_G_ value, the fewer defects of the carbon atoms in the formed structure, and the higher the degree of graphitization of the charred layer, indicating a better quality of the combustion residue of the material [35,40]. A good stable carbon residue can act as a barrier to isolate the combustible medium from air at high temperatures [38,39]. The I_D_/I_G_ of (PP/IFR)/SF, (PP/SF)/IFR, and PP/IFR/SF was 0.69, 0.61, and 0.63, respectively. The I_D_/I_G_ of (PP/SF) /IFR composites was the lowest, which indicated that the degree of graphitization of the carbon residue was the highest. 

### 3.3. Thermal Stability of PP/SF Composites

**Figure 6:**
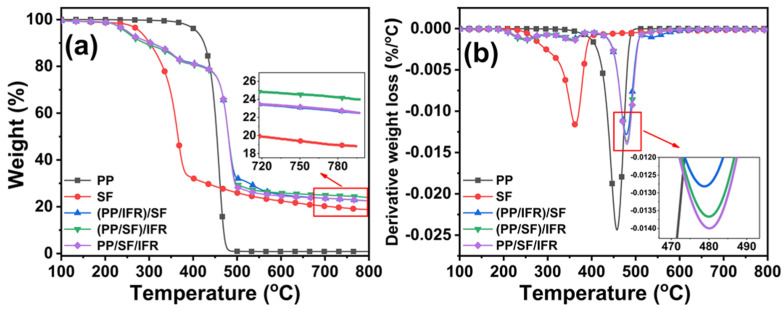
TGA (**a**) and DTG (**b**) of PP, SF, and PP/SF flame−retardant composites.

The fire resistance of materials is closely related to the thermal stability of materials, which can be obtained using thermogravimetric analysis. Figure 6 and Table 3 are the TG and DTG results of PP, SF, and PP/SF flame-retardant composites. PP was thermally stable, with an initial thermal decomposition temperature (T_−5%_) as high as 409.5 °C; nonetheless, there was little residue after thermal decomposition at 800 °C. The sisal fibers were thermal-sensitive when the temperature was above 200 °C [41], and the T_−5%_ of sisal fiber was only 269.1 °C; however, the sisal fiber did have a high residue of 18.3% at 800 °C, which means that sisal fiber can be used as a carbon forming agent to enhance the flame retardation of the condensed phase of PP [2]. The introduction of flame retardants further reduces the thermal stability of composites, which is related to the action mechanism of flame retardants. In the case of fire, the thermal decomposition of flame retardants occurs before the substrate, so as to produce the corresponding chemical substances to inhibit the combustion process of the matrix and play the role of flame retardation. The thermal degradation trend of the three kinds of PP/SF flame-retardant composites was consistent. There were slight differences between the initial decomposition temperatures of (PP/SF)/IFR, (PP/IFR)/SF, and PP/IFR/SF composites, where T_−5%_ of the (PP/SF)/IFR composite was the lowest, which was 245.6 °C. However, the temperatures of 50% thermal weight loss (T_−50%_) and the maximum weight loss (T_max_) of composites obtained under different processing sequences were similar. Additionally, the char yield of the (PP/SF)/IFR composite at 800 °C was 23.9%, slightly higher than the other two materials. 

### 3.4. Flame-Retardant Mechanism

**Figure 7:**
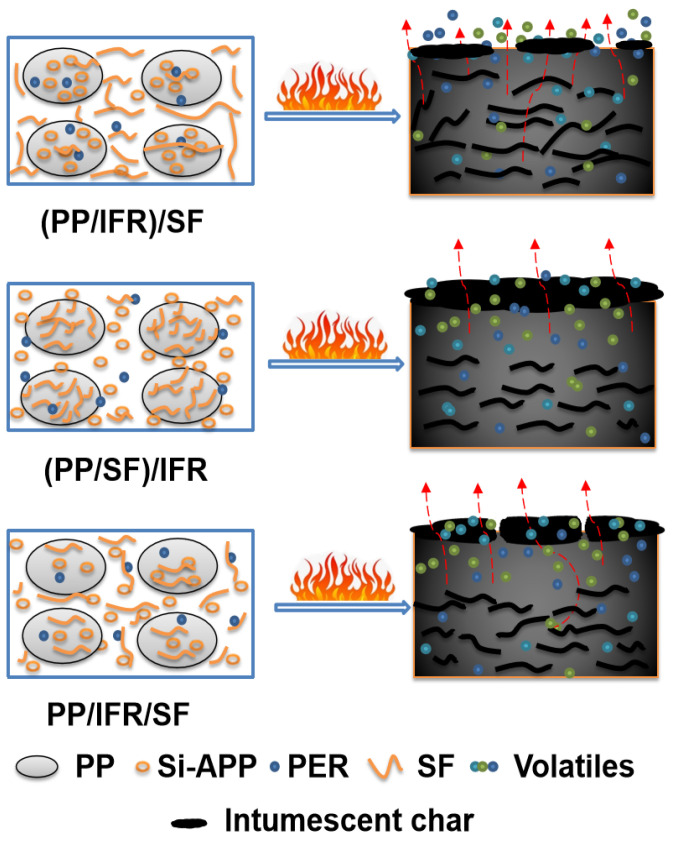
Possible flame-retardant mechanism of PP/SF flame-retardant composites.

The flame-retardant mechanism that may be involved in the combustion process of the PP/SF flame-retardant composites is shown in Figure 7. During the combustion process, Si-APP catalyzed the carbon formation of sisal fiber and the PP matrix, along with the release of some volatiles from the decomposition of the substrate. In the (PP/IFR)/SF composites, the matrix was first blended with flame retardants. In this process, the flame retardants were coated by the matrix, and then blended with fiber in the secondary extrusion process, and most of the flame retardants were separated from the fibers by the matrix; less were attached to the surface of the fibers. This resulted in poor carbonization of the fiber surface during combustion, as shown in Figure 4a. The bare fiber interface provided a channel for the transport of oxygen and combustibles during the combustion process, resulting in a poor flame-retardant effect of the composites. In addition, the fibers in the (PP/IFR)/SF composite underwent only one extrusion process and had poor dispersion in the matrix; this resulted in an uneven charred layer formed during combustion and obvious cracks on the surface of the carbon layer, as shown in Figure 3a, which led to the deterioration of the flame-retardant performance of the composite. In the (PP/SF)/IFR composites, PP was blended with SF first, the fiber was wrapped by PP, and then the flame retardants were wrapped in the outer layer of PP. During the combustion process, the flame retardant decomposed in advance, promoting the carbonization of the internal matrix and forming a dense carbon layer on the fiber surface, as shown in Figure 3b and Figure 4b. The charred layer with the expansion structure formed during combustion can effectively protect the base from thermal decomposition, endowing the composite with good flame-retardant performance. Polypropylene, fiber, and flame retardants in the PP/IFR/SF composite were randomly dispersed, and the flame-retardant effect was relatively poor.

### 3.5. Mechanical Properties

**Figure 8:**
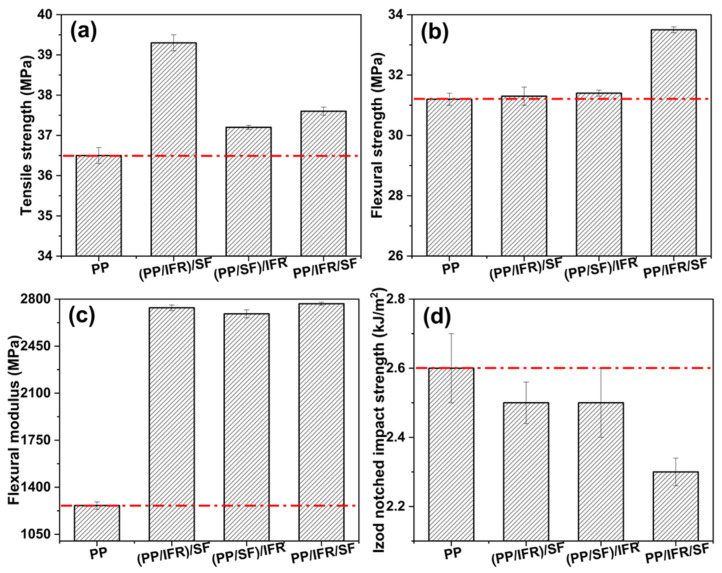
Mechanical properties of pure PP and PP/SF flame-retardant composites. (**a**) Tensile strength, (**b**) Flexural strength, (**c**) Flexural modulus, (**d**) Izod notched impact strength.

The effects of different mixing sequences on the mechanical properties of the PP/SF flame-retardant composites are shown in Figure 8. In general, the addition of fiber and flame retardants improved the tensile and flexural properties of PP, while they deteriorated the notched impact strength of PP, which was due to the introduction of fibers and flame-retardant particles increasing the defects of the system, leading to higher stress concentrations and therefore the failure of the material at a lower stress.

The flexural strength and modulus (Figure 8) of the composites showed an increase with the incorporation of sisal fibers and flame retardants in the pure PP. Flexural strength represents the load-carrying capacity of the specimen, which is related to the distribution of the fillers in the composite and the interfacial adhesion between fillers and the matrix. Both the flame retardants and fibers in the PP/IFR/SF composite underwent secondary blending and were well dispersed in the matrix, endowing PP/IFR/SF a better bending strength than that of (PP/SF)/IFR and (PP/IFR)/SF. The flexural moduli of the obtained composites were of the same order of magnitude with pure PP. As is well known, various mechanisms such as shearing, tension, and compression take place simultaneously during the bending test [42]. The failure characteristics of composites were completely changed as a result of the addition of fibers and flame-retardant particles. Moreover, the modulus of the added natural fiber was comparatively lower than that of the inorganic fibers such as glass fiber and carbon fiber; therefore, a combination of influencing factors leading to the composite modulus is not an order of magnitude higher than that of pure PP. Similar results have been observed by some research groups [5,42,43].

Compared with the (PP/IFR)/SF and PP/IFR/SF systems, the overall mechanical properties of the (PP/SF)/IFR composites were slightly worse. The tensile strengths of the PP/IFR/SF and (PP/SF)/IFR composites were relatively low, especially that of the (PP/SF)/IFR composites, which was 37.2 MPa. In general, in a certain size range, the longer the fiber length in the fiber-reinforced products, the more damage work the fiber consumes through its own fracture in the process of material failure, and thus the higher the mechanical strength of the composite material [44,45]. Therefore, the (PP/IFR)/SF composite exhibits the best mechanical properties. However, after secondary extrusion shearing, the length of the fibers in the PP/IFR/SF and (PP/SF)/IFR composites was obviously damaged, which led to the reinforcement effect of the fiber being obviously weaker than that of the (PP/IFR)/SF composites.

**Figure 9:**
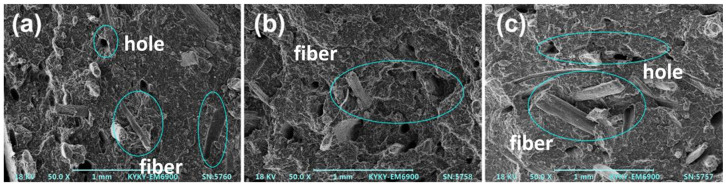
SEM micrographs of PP/SF flame-retardant composites. (**a**) (PP/IFR)/SF; (**b**) (PP/SF)/IFR; (**c**) PP/IFR/SF.

The interfacial bonding and fiber dispersion within the fiber-reinforced polymeric composites can be evaluated by the morphology of the impact section. The SEM micrographs of the impact section of the PP/SF composites are shown in Figure 9. As can be seen from the figure, the fibers of composite materials obtained by different blending processes had different degrees of pulling out when the samples were destroyed by external forces. As shown in the position indicated by the ellipse in Figure 9a, fiber pull-out in (PP/IFR)/SF was obvious, leaving many pulled-out marks and holes. There was little resin matrix residue on the surface of the pulled-out fiber. However, there was no obvious peeling phenomenon between the fiber and matrix in Figure 9b and c, which indicated that the interface bonding between the fiber and the matrix in (PP/SF)/IFR and PP/IFR/SF was better than that of (PP/IFR)/SF. This also showed that the secondary blending of the fiber and matrix was conducive to the interfacial bonding between the fiber and matrix. Moreover, it can be seen that the length of the fiber pulled out in the (PP/SF)/IFR sample was shorter than that of the PP/IFR/SF sample, indicating that the interfacial bonding between the fiber and the matrix of (PP/SF)/IFR was better than that of PP/IFR/SF, showing a slightly higher notched impact strength of 2.5 kJ/m^2^.

## 4. Conclusions

Sisal-fiber-reinforced polypropylene flame-retardant composites were prepared via twin-screw extrusion and the effects of different blending procedures on the fire retardancy and mechanical performance of the composites were investigated. In the case of the same raw material formula, the (PP/SF)/IFR composite prepared by PP, first blended with SF, and then with flame retardants for secondary blending, showed an excellent flame-retardant performance, with a limited oxygen index of 28.3% and reaching a UL-94 V-0 rating. The tensile strength of the (PP/SF)/IFR system was 5.3% lower than that of the (PP/IFR)/SF system; however, the comprehensive mechanical properties of (PP/SF)/IFR were not much different from those of the composites prepared via the other two mixing processes. It is clear from the present study that the blending procedure has a great influence on the properties of the composites, especially the flame-retardant properties. A useful composite with good fire resistance and strength could be successfully developed through the process of matrix blending with fiber first, and then secondary blending with flame retardants. These good properties allow sisal-fiber-reinforced polypropylene flame-retardant composites to exhibit great application prospects in construction and the automotive industry.

However, adding large amounts of flame retardants is still the main means to realize the flame retardancy of existing natural-fiber-reinforced polymeric composite products, which will undoubtedly lead to the deterioration of the mechanical properties of the composites, especially the notched impact properties as shown in this study. In order to balance the flame retardancy and mechanical properties of nature fiber-reinforced polymeric composites, some key technical problems such as the compatibility between the matrix and fillers, the multiple interfacial processing technologies between fiber, matrix, and flame retardants, and the interfacial heat conduction problem need to be solved. From a practical and commercial point of view, the majority of scientific research workers and enterprises still need to commit to developing halogen-free, low-smoke, low-toxicity flame retardants and new methods of flame retardant modification with high efficiency and environmental protection, simplifying the natural fiber flame retardancy treatment process, and ultimately realizing the development of high efficiency halogen-free flame retardants and high-performance natural-fiber-reinforced polymeric flame-retardant composites.

## Figures and Tables

**Table 1:** Formulation and UL-94 rating, as well as limiting oxygen index of original PP and PP/SF composites.

Designation	PP (wt%)	PER (wt%)	Si-APP (wt%)	SF (wt%)	Auxiliaries (wt%)	UL-94 (3.2 mm)	LOI (%)
PP	95	0	0	0	5	NR ^a^	18.4
(PP/IFR)/SF	45	7.5	22.5	20	5	NR	24.7
(PP/SF)/IFR	45	7.5	22.5	20	5	V-0 ^b^	28.3
PP/IFR/SF	45	7.5	22.5	20	5	NR	25.7

^a^ No rating. ^b^ A single sample could be extinguished within 10 s after double fire ignition, and the total flame burning time of five samples was not more than 50 s.

**Table 2:** Cone calorimetric combustion experimental results of pure PP and PP/SF flame-retardant composites.

Designation	PP	(PP/IFR)/SF	(PP/SF)/IFR	PP/IFR/SF
TTI (s)	36	23	29	28
pk HRR (kW/m^2^)	646.2	362.2	321.3	328.6
THR (MJ/m^2^)	135.3	141.6	122.2	134.9
mean EHC (MJ/kg)	25.2	22.3	21.3	22.0
SEA (m^2^/kg)	299.3	451.0	477.3	461.6
Av CO (kg/kg)	0.02	0.08	0.09	0.07
Av CO_2_ (kg/kg)	1.4	1.2	1.1	1.1
TSP (m^2^)	14.3	25.3	24.1	24.9

TTI: Time to ignition; pk HRR: Peak heat release rate; THR: Total heat release; mean EHC: Mean effective heat of combustion; SEA: Specific extinction area; Av CO: Average carbon monoxide yield; Av CO_2_: Average carbon dioxide yield; TSP: Total smoke production.

**Table 3:** Thermal analysis data of PP/SF flame-retardant composites.

Designation	T_−5%_/°C	T_−50%_/°C	T_max_/°C	Residue/%
PP	409.5	455.3	492.5	0.8
SF	269.1	363.6	361.8	18.3
(PP/IFR)/SF	249.0	480.4	479.0	22.5
(PP/SF)/IFR	245.6	480.2	480.1	23.9
PP/IFR/SF	250.6	480.0	480.4	22.5

## Data Availability

Available data can be obtained from the corresponding author upon request.
